# Human Resources for Health: The Best Learning, the Best Skill Mix, and the Most Impact

**DOI:** 10.9745/GHSP-D-18-00092

**Published:** 2018-03-21

**Authors:** James D Shelton

**Affiliations:** a Editor-in-Chief Emeritus, Global Health: Science and Practice Journal, Baltimore, MD, USA.

## Abstract

Acting in a difficult environment, constructive efforts to improve medical education in Zimbabwe included revised curricula, investing in faculty and improved teaching skills, competency-based learning, and modern technology. But an ideal approach to health systems strengthening would put more emphasis on primary care and prevention, equity, and the many other vital health cadres besides physicians.

See related article by Hakim et al.

We have witnessed markedly increased attention to the health workforce in low- and middle-income countries (LMICs) in the last 2 decades. And with good reason. Health workers are the backbone of all health systems, and the situation is generally dire. A good write-up is found in the recent *Lancet* Commission on the future of health in sub-Saharan Africa.^1^ Some key themes:

- Huge need for additional staff
- Priority for primary care and prevention
- Priority for a diverse set of health cadres
- Equity for underserved populations
- Competency-based learning
- Information technology for learning
- Institution strengthening and sustainability, including lifelong learning
- Collaboration across countries—of both pedagogic and technical content

Addressing all this is of course a very tall order. In this issue of GHSP, we feature one helpful step addressing this immense challenge at the preservice level. Hakim and colleagues^2^ describe their efforts to strengthen medical education at the University of Zimbabwe, under the auspices of the Medical Education Partnership Initiative (MEPI) funded by the U.S. President's Emergency Plan for AIDS Relief (PEPFAR) at $13 million over 5 years. PEPFAR is to be commended for undertaking this initiative, consistent with the proposition that overall health systems strengthening will be necessary for HIV control to be sustainable in the long term.^3^

## AT ITS FOUNDATION: A DEFINITE STRATEGY AIMED AT IMPROVING THE MEDICAL EDUCATION SYSTEM

First, recognize that medical education in Zimbabwe had been in serious disarray following a series of economic crises. Under those constraints, as described in their article, Hakim and colleagues creditably focused on a number of the areas listed in the bullets to the left. These included strengthening and reforms at the medical school level, such as revising curricula; implementing competency-based learning approaches; improving faculty teaching skills, including an intensive faculty development and scholarship program; recruiting new faculty; increasing student enrollment; improving use of modern digital technology in learning; and collaborating with international partners.

### Limited Ability to Assess Both Process and Outcomes

Still, Hakim and colleagues provide only limited evidence for clear effectiveness of their efforts. Findings presented are mainly derived from surveys of participants, such as in workshops. Thus, we are given little documentation of what actually happened in trainings and no objective assessment of improvements in knowledge or practice. Moreover, the numbers of respondents reported in the surveys are often rather modest, limiting interpretability. Findings from the surveys are generally positive, though such surveys can, of course, be highly subject to courtesy bias.

The authors do present some statements about outcomes but without clear substantiation. For example, regarding the new HIV curriculum, they assert that its effects “… were noticed in the main teaching hospitals first, and then extended out as doctors and specialists became more qualified and spread to other parts of the country.” But supporting evidence or elaboration is not provided. They also point to a marked increase in numbers of practitioners in Zimbabwe during this time period. But the number of new graduates is not nearly enough to account for this large increase. Rather, the main explanation appears to be returning physicians who had previously left the country during times of extreme economic and political stress. And because of the limited time scope of the article, we have little sense of potential long-term effects.

### Shortcomings of the Approach: Level and Equity of Interventions and Range of Cadres

The fundamental goal for any health system should be to have the most impact to improve health across the population. That should include emphasis on primary care and prevention including healthy lifestyle and structural prevention interventions like sanitation. Such needs are especially great in LMICs like Zimbabwe. The effort clearly did focus on improving HIV care and the article includes a passing reference to social determinants of health. Yet the program succumbed very substantially to the seemingly inevitable drive in medical culture to gravitate toward subspecialty care and technical procedures to include components like forensic psychiatry, pacemaker implantation, electroencephalography for epilepsy patients, and bronchoscopy.

Health systems should place emphasis on primary care and prevention.

Likewise, the objective of equity calls for particular efforts toward interventions directed to rural areas, where few physicians reside, as well as toward the most needy in the population. Unfortunately, there is only some very limited description of “community-based education” and efforts to improve it, and little discernable specific attention to reaching the neediest.

The objective of equity calls for particular efforts toward interventions directed to rural areas and to the most needy in the population.

Lastly, all health systems need many other categories of health professionals besides physicians. Indeed, in resource-poor countries, optimal provision of services calls for even more emphasis on less-trained categories of health workers. Physicians arguably should not even be the first priority. While MEPI had a collateral effort to support nursing (NEPI), the *Lancet* Commission rightly emphasizes the importance of task shifting and task sharing and support for many other cadres. The long list of other key cadres, which often get insufficient attention, include clinical officers, community health workers, pharmacists and pharmacy assistants, midwives, sanitarians, social workers, lab technicians, emergency technicians, anesthesia technicians, logistics specialists, epidemiologists, safety engineers, computer technologists, behavioral scientists, and managers.

MEPI was obviously created to focus on physicians, so the authors needed to work within that framework. But physicians have an important role in supporting and aligning with these other cadres. This facilitating aspect was not addressed by the authors.

## CONCLUSION

Rome was not built in a day. And the perfect should not be the enemy of the good. The efforts by Hakim certainly appear to have moved in the direction to improve the health system in Zimbabwe. But what is really needed in LMICs in general is a broader, more complete approach.
